# Thrombus-Targeting Polymeric Nanocarriers and Their Biomedical Applications in Thrombolytic Therapy

**DOI:** 10.3389/fphys.2021.763085

**Published:** 2021-11-30

**Authors:** Qixiao Guan, Hongjing Dou

**Affiliations:** State Key Laboratory of Metal Matrix Composites, School of Materials Science and Engineering, Shanghai Jiao Tong University, Shanghai, China

**Keywords:** thrombosis, polymeric nanocarriers, targeted delivery, biomimetic technology, antithrombotic strategies

## Abstract

Due to the high morbidity and mortality of cardiovascular diseases, there is an urgent need for research on antithrombotic strategies. In view of the short half-life, insufficient drug penetration, poor targeting capabilities, and hemorrhagic side-effects of traditional thrombus treatment methods, the combination of thrombolytic therapy and nanocarriers brought by the development of nanotechnology in recent years may provide effective solutions for these undesirable side-effects caused by insufficient targeting. Polymeric nanocarriers, based on macromolecules and various functional groups, can connect specific targeting molecules together through chemical modification to achieve the protection and targeted delivery of thrombolytic drugs. However, simple chemical molecular modifications may be easily affected by the physiological environment encountered in the circulatory system. Therefore, the modification of nanocarriers with cell membranes can provide camouflage to these platforms and help to extend their circulation time while also imparting them with the biological functions of cell membranes, thus providing them with precise targeting capabilities, among which the most important is the biological modification of platelet membranes. In addition, some nanoparticles with their own therapeutic functions have also been developed, such as polypyrrole, which can exhibit a photothermal effect to induce thrombolysis. Herein, combined with the mechanism of thrombosis and thrombolysis, we outline the recent advances achieved with thrombus-targeting nanocarriers with regard to thrombosis treatment. On this basis, the design considerations, advantages, and challenges of these thrombolytic therapies in clinical transformation are discussed.

## Introduction

At present, cardiovascular disease is still one of the most threatening diseases to human health and life in the world. Cardiovascular diseases have a high morbidity and mortality rate. According to statistics, approximately 179 million people die from cardiovascular diseases each year, accounting for about 31% of the world’s death toll, and this number is anticipated to reach over 236 million by 2030 (Benjamin et al., 2020; Zenych et al., 2020). There are three main types of cardiovascular diseases, including myocardial infarction, cerebral stroke, and deep vein thrombosis. These three cardiovascular diseases are primarily caused by thrombosis, which is a blood clot blockage of blood vessels (Fuster et al., 1992; Gao et al., 2021; Rodriguez and Harrington, 2021; Xue et al., 2021). Therefore, the prevention and treatment of thrombosis are an urgent issue, and it is also of great research significance.

Currently, the most important remedy for thrombosis and related cardiovascular diseases is prevention, but in cases involving long-term thrombosis, the main treatment options include balloon catheterization, surgical embolectomy, thrombolytic therapy, and other relevant surgeries (Powers et al., 2018). Taking into account the cost of surgical treatment and the damage it causes to the body, the application of thrombolytic drugs has increasingly become an important strategy. Thrombolytic agents include tissue plasminogen activators (tPA), recombinant tissue plasminogen activators (rtPA), urokinase (UK), streptokinase (SK), and other plasminogen activators (PAs; Marder, 2013; Cheng et al., 2018; Zamanlu et al., 2018; Ma et al., 2019; Hassanpour et al., 2020). The diverse antigenicity, half-life, lytic potential, fibrin specificity, and hemorrhagic risks associated with these agents lead to their differing effectiveness (Marshall, 2015). In addition, anticoagulants and antiplatelet drugs are often used to prevent clotting (Bala et al., 2018; Al Rawahi et al., 2019). These drugs often have only limited effectiveness when they are used alone, for example, their penetration into clots tends to be trivial and requires larger doses. Making matters worse, they may also cause hemorrhagic transformation leading to fatal intracerebral hemorrhage, as well as some other undesirable side-effects (Pfefferkorn and Rosenberg, 2003; Mao et al., 2017; Powers et al., 2018; Xu et al., 2021).

**Figure 1:**
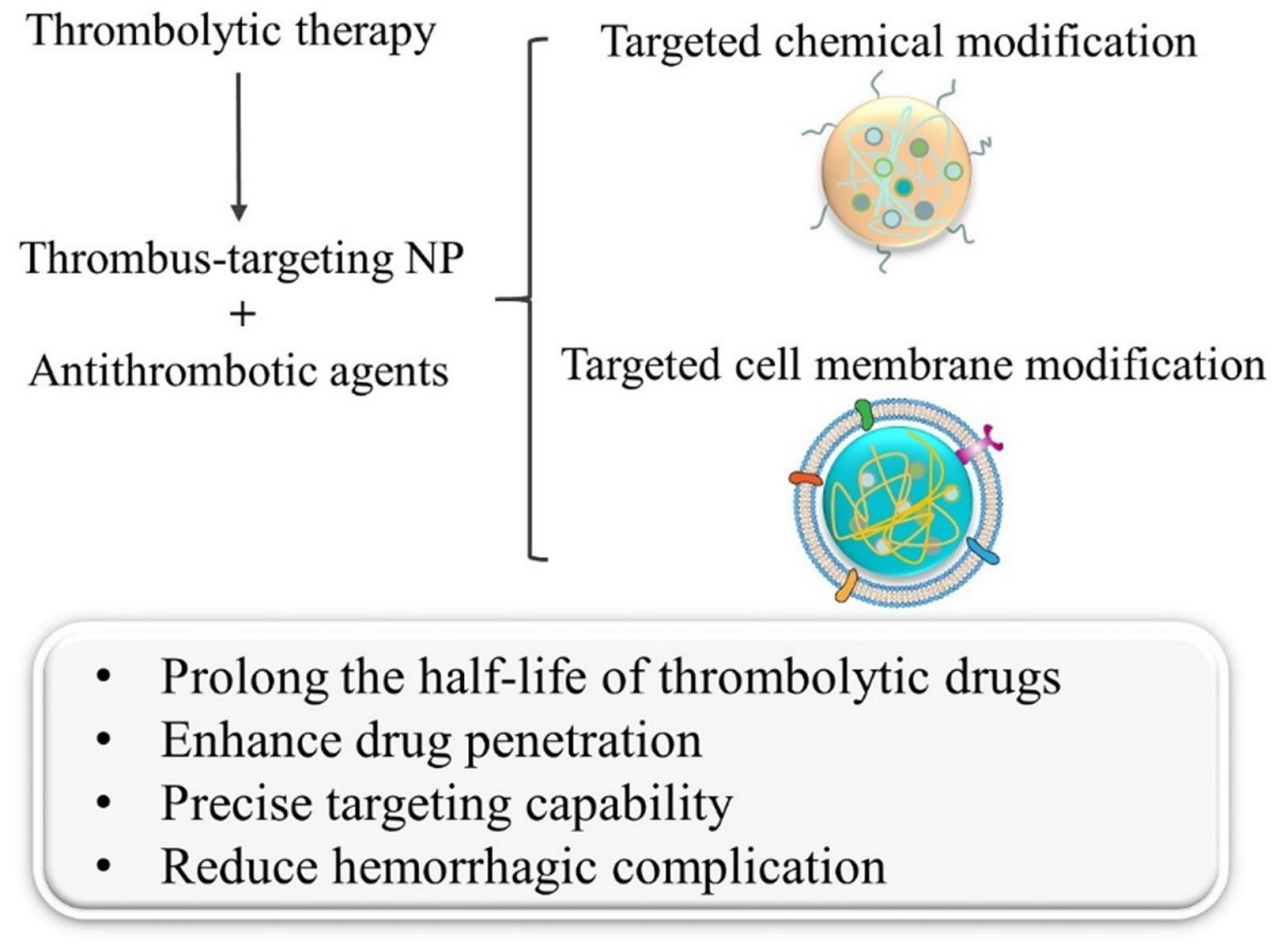
Schematic diagram of the two main modification methods in thrombus-targeting nanoparticles and their effects in thrombolytic therapy.

With the rapid development of nanotechnology and biotechnology (Jiang et al., 2020; Cornel et al., 2021), the combination of thrombolytic therapy and nanocarriers may provide a novel solution to key issues, such as the short half-life, low targeting ability, and unexpected bleeding complications of the existing therapeutic drugs (Zamanlu et al., 2018; Ma et al., 2019; Hassanpour et al., 2020). Polymeric nanocarriers based on macromolecules can provide the protection and targeted delivery of thrombolytic drugs through the unique physicochemical property brought by the nanoscale and the facile design of polymer chains. In addition, biologically inspired cell membrane modification strategies can transfer the specific functions and biological characteristics of cell membranes to the nanocarriers, thus improving the biocompatibility and precise targeting ability, and greatly enhancing the effect of thrombolytic therapy (Figure 1).

## Mechanism of Thrombosis and Thrombolysis

Thrombosis is primarily triggered by collagen, tissue factor, thrombin, and other factors, which induce local platelet activation and fibronectin complex formation (Zenych et al., 2020). These key components jointly participate in the construction of the intravascular microenvironment that can lead to thrombosis and promote cancer. Fibrinogen is an important reaction substrate for thrombosis and is the key step involved in thrombosis (Undas and Ariens, 2011). When the endogenous coagulation system is abnormally activated, fibrinogen will form fibrin monomers under the action of thrombin, activated factor XIII, Ca^2+^, and other coagulation factors, subsequently covalently binding with each other to form a fibrin polymer. This stable fibrin network finally captures red blood cells, platelets, α2-antiplasmin, and other components to form a stable thrombus structure (Chernysh et al., 2011; Alkarithi et al., 2021).

A thrombus has a complex structure and composition, and notably, it bears numerous targetable receptors for nanocarriers that can themselves carry various ligands. By modifying the surfaces of nanocarriers with targeting moieties (such as antibodies, aptamers, peptides, and cell membrane proteins; Li et al., 2020), they can selectively target various biomarkers (P-selectin, Integrin GPIIb/IIIa, Factor XIII, and fibrin) at the thrombus site, thus achieving specific thrombus-targeting capabilities and enhancing the therapeutic effect due to the accumulation of thrombolytic drug at the surface of a clot (Karagkiozaki et al., 2015; Zenych et al., 2020). As far as the modification of the platelet membrane is concerned, the targeting mechanism may proceed *via* the upregulation of GP-Ib-V-IX glycoprotein complex and integrin GPIIb/IIIa residing on the surface of platelet membrane which in turn promotes the adhesion of fibrin and collagen, thus enabling the nanocarrier to target thrombi (Doshi et al., 2012; Chang et al., 2015).

**Figure 2:**
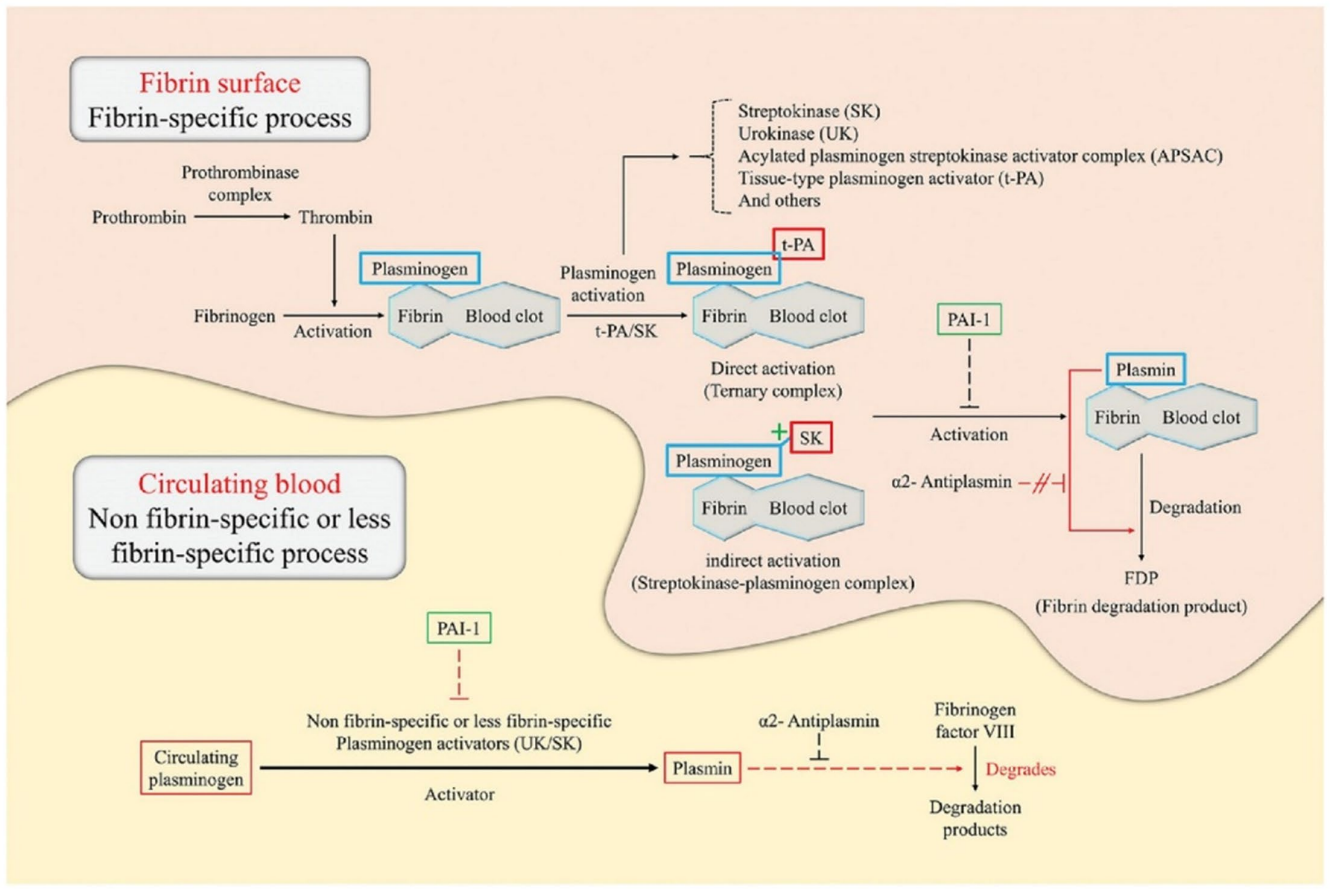
Illustration of the principles of thrombolysis in a fibrin surface and circulating blood environment (Hassanpour et al., 2020). This figure describes the catalytic principle of the conversion of plasminogen to plasmin according to the binding method of the plasminogen activator [e.g., tissue-type plasminogen activator (tPA), urokinase (UK), and streptokinase (SK)]) Plasminogen specifically binds to the surface of the fibrin blood clot. In direct activation, tPA preferentially attaches to plasminogen, resulting in the formation of a ternary complex. On the other hand, in indirect activation, SK cannot directly bind to the plasminogen but induce conformational changes of the plasminogen to form a streptokinase-plasminogen complex. Subsequently, these complexes form plasmin through cleavage of the fibrin-associated plasminogen. Plasmin formed by direct/indirect activation breaks down fibrin into fibrin degradation products, which eventually dissolves blood clots. The thrombolytic process in circulating blood is triggered by non-fibrin-specific or less fibrin-specific plasminogen activators. Plasminogen activators, such as the UK and SK, induce plasmin production by cleavage of circulating plasminogen. Subsequently, plasmin degrades fibrinogen factor VIII instead of fibrinogen. Plasmin activator inhibitor-1 acts on plasminogen, blocking cleavage into plasmin, and causing blood clot formation. α2-antiplasmin acts only on circulating blood and can inhibit thrombolysis by interfering with plasmin binding sites with fibrinogen factor VIII.

In addition, the proportion of platelets is higher in the case of arterial thrombosis, while venous thrombosis contains more red blood cells and a denser fibrin network (Mackman, 2008; Martinelli et al., 2010). Consequently, existing strategies for thrombus treatment are primarily aimed at platelets and fibrin networks. For example, when the nanocarriers are targeted to reach the thrombus site, the plasminogen activator that they release can activate the plasminogen near the thrombus to become plasmin (Altaf et al., 2021), and then dissolve the cross-linked bonds between fibrin, destroy its network structure, and thus dissolve the blood clot (Figure 2; Collen and Lijnen, 2005; Hassanpour et al., 2020).

## Targeted Chemical Modification of Nanocarriers and Applications

The development of antithrombotic therapy has been a high priority for many years (Zhao et al., 2020a). Owing to the rapid progression of nanotechnology, new antithrombotic nanotherapeutics have sprung up in recent years. The nanoscale characteristics of nanocarriers can introduce unique physical and chemical properties, and extend the circulation time of drugs in the bloodstream (Wang et al., 2018a; Zhang et al., 2018). Moreover, the long-chain structure of polymer macromolecules also facilitates the modification of these platforms for targeting capability and enables researchers to design nanocarriers and impart them with specific functions in order to meet a given requirement (Guo et al., 2018; Wang et al., 2018b; Shakiba et al., 2021). Therefore, the design, modification, and application of polymeric nanocarriers for thrombolytic therapy have become a significant research focus in recent years (He et al., 2021).

The upregulation of integrin GPIIb/IIIa on the surfaces of activated platelets is considered to be an important sign of thrombosis (Bai et al., 2020). The ligand corresponding to this integrin is known to be cyclic arginine-glycine-aspartic acid (cRGD; Barre, 2007; Ye et al., 2020). Therefore, Huang et al. reported an activated-platelet-sensitive nanocarrier capable of inducing selective thrombolysis through targeted delivery and controlled release of tPA to blood clot (Huang et al., 2019). The nanothrombolytic system loaded with tPA was based on PEGylated liposome, which can provide better *in vivo* stability. The incorporation of cRGD on the surface of liposome is the source of activated platelet sensitivity, it has been reported that cRGD peptide has high specificity and affinity for integrin GPIIb/IIIa on activated platelets (Ye et al., 2020).

Several studies have demonstrated that cRGD-modified nanosystems can effectively induce the selective lysis of fibrin and blood clots, thus suggesting that they are promising candidates for targeted thrombolytic therapy (Li et al., 2019; Zhang et al., 2019; Zhong et al., 2020). However, it is worth noting that studies have shown that platelet activation primarily occurs during the early stage of thrombosis, and the influence of integrin GPIIb/IIIa on platelet aggregation and thrombosis also decreases with the progression from the early to the late stage of the thrombosis process (Yan et al., 2000; Zhou et al., 2011; Gambino et al., 2021). In other words, the treatment brought by cRGD-modified GPIIb/IIIa-targeting nanocarriers primarily targets the early stages of thrombosis. So in practical applications, attention should thus be paid to the specific development stage of thrombosis and suit the right medicine to the case. In addition to the above systems, an organic semiconducting nanoparticle has also been chemically modified with cRGD peptides for use as a photoacoustic (PA) contrast agent for selectively reducing of early thrombosis (Cui et al., 2017), and the addition of the PA signal enhancing molecules can achieve specific imaging and lysis of blood clots (Wang et al., 2021).

Injection of rtPA is the standard drug treatment for thrombolysis (Hassanpour et al., 2020). However, due to the short half-life of rtPA, it only exhibits limited therapeutic efficiency even at high doses, and it brings a higher risk of bleeding, resulting in a 6% intracranial hemorrhage rate and a 50% chance of subsequent mortality (Yaghi et al., 2014). Therefore, polysaccharide-poly(isobutylcyanoacrylate) nanoparticles functionalized with fucoidan and loaded with rtPA were designed to accumulate on the surface of a thrombus (Juenet et al., 2018). Low-molecular weight fucoidan has a nanomolar affinity for P-selectin, which is expressed by activated platelets in thrombus (Bachelet et al., 2009; Zenych et al., 2021). Positively charged aminated dextran was incorporated into the polysaccharide shell to promote electrostatic interactions (Mukwaya et al., 2019), thereby mimicking the natural fibrin binding sites. The experimental results emphasized the relevance of targeting P-selectin for the treatment of thrombosis with rtPA for the first time, which is of great significance for the subsequent development of nanoparticles with P-selectin as the target of thrombosis treatment. But in the actual treatment process, it is best to use it in combination with other targets, because P-selectin can also be used as a target in tumor treatment (Farokhzad, 2015).

By taking into account of the biological characteristics of thrombosis, including the upregulation of H_2_O_2_ and the abundance of fibrin (Dayal et al., 2013; Andreadou et al., 2021), Zhao et al. (2020b) have developed a H_2_O_2_-responsive nanocarrier for the thrombus-targeting delivery of the antithrombotic agent tirofiban (Zhao et al., 2020b). This nanocarrier was composed of a dextran nanocore and a red blood cell (RBC) membrane shell, and tirofiban was conjugated to dextran through a H_2_O_2_-cleavable phenylboronic ester linkage. In contrast with the previous study on the nanosystem modified by fusion protein CREKA and H_2_O_2_-scavenging boronate group (Kang et al., 2017), the coating of the RBC membrane can enhance the circulation ability *in vivo*, and the functionalized peptide CREKA on RBC membrane can provide the desired thrombus-specific targeting capability. The ingenuity of the structural design in this study is that it can not only use the H_2_O_2_ to cleave the phenylboronic ester linkage to release thrombolytic drugs, but also effectively scavenges H_2_O_2_ and protects cells against H_2_O_2_-induced cytotoxicity. It is noteworthy that the platelet membrane may be more suitable for thrombus-targeting than the RBC membrane due to its unique thrombus-homing property, but tirofiban is likely to compromise the receptor on platelet membrane and thus may weaken the targeting capability (Chang et al., 2015). This work can also consider strengthening the research on the use of erythrocyte membrane to overcome barriers in biological microenvironments (Castro et al., 2021).

In addition, some nanoparticles with their own therapeutic functions have also been developed to utilize the photothermal effect as a means to achieve thrombolysis (Burnouf et al., 2019; Huang et al., 2020; Yuan et al., 2021). In order to avoid side-effects, such as hemorrhagic risk, that are generally associated with thrombolytic drugs, near-infrared light-mediated photothermal thrombolysis has been developed as a new treatment for thrombus (Ghosh and Pal, 2007; Singh et al., 2016). Following the initial work by Singh et al. (2016) and Chuang et al. (Mi et al., 2017; Satapathy et al., 2018), a new thrombolytic therapy utilizing photothermal decomposition of fibrin clots was explored by using dual-targeting glycol chitosan/heparin-modified polypyrrole nanoparticles to enhance targeted delivery and thrombolytic effect (Lu et al., 2021). Among them, glycol chitosan showed specific self-adaptive targeting capabilities in the acidic microenvironment of pathologically inflamed tissues at the thrombus (Lee et al., 2014), and heparin had potential biological affinity toward P-selectin that is highly expressed at the thrombus (Ludwig et al., 2004; Schwarz et al., 2020) and finally achieved high-efficiency thrombolysis by leveraging the photothermal effect exhibited by polypyrrole. In contrast with traditional thrombolytic therapy, this novel approach used external near-infrared light to cooperate with the nanoparticles delivered into the body to achieve thrombotic therapy and has promising prospects in the field of thrombolytic therapy.

## Targeted Natural Cell Membrane Modification of Nanocarriers and Applications

Although great progress has been made with chemically modified polymeric nanocarriers with regard to targeted thrombolytic therapy, simple chemical molecular modification may be easily affected by the physiological environment that is encountered during the blood circulation process, thus resulting in inactivation of targeting molecules, agglomeration and adhesion of nanocarriers, and other issues. To address these issues, biologically inspired polymeric nanocarriers which were modified by cell membrane have been developed. This modification strategy serves to camouflage the nanocarriers and provides an extended circulation time, while also imparting the nanocarriers with the biological functions of natural cell membranes, so as to achieve more precise targeting capabilities (Xuan et al., 2019; Guan et al., 2021).

Inspired by the innate roles of platelets in hemostasis and pathological thrombus (Falati et al., 2002; Lippi et al., 2011), platelet membrane-camouflaged polymeric nanoparticles (nanoplatelets) have been developed to enable the targeted delivery of thrombolytic drug to local thrombus sites (Hu et al., 2015). In a recent study, Xu et al. (2020) bound a platelet membrane to the surface of poly(lactic-*co*-glycolic acid; PLGA) polymeric inner cores, and rtPA was then chemically conjugated to the activated sulfhydryl groups residing on the external surface of the platelet membrane to form PNP-PA (Xu et al., 2020). It was found that PNP-PA possesses the major membrane adhesion-associated proteins, which can be used to achieve targeted thrombolysis. In addition, researchers also determined the two most effective receptors on activated platelets for PNP-PA recruitment, namely, GPIIb/IIIa and P-selectin mentioned above. Furthermore, the analysis of *in vivo* coagulation indicators in different thrombosis models suggested that the nanoplatelets exhibit a low risk of bleeding complications. Therefore, this nanoplatelet strategy offers an integrated solution to address the drawbacks of clinically used thrombolytic drugs and has great potential to refine the current state of thrombosis treatment.

Using a conceptually similar strategy, Wang et al. (2020) developed platelet membrane-coated PLGA cores loading lumbrokinase as nanoplatelets (PNPs/LBK) to achieve effective thrombolysis with reduced hemorrhagic risk (Wang et al., 2020). Through platelet membrane coating, the circulation time of PNPs is as long as that of RBC membrane-coated nanoparticles. After the PNPs were anchored into the thrombus site through heteromultivalent ligand-mediated binding to active platelet integrin GPIIb/IIIa and P-selectin, the thrombolytic payload was released due to vesicle destabilization triggered by clot-relevant enzyme phospholipase-A2 (Pawlowski et al., 2017). Importantly, hemorrhagic tests reveal that the administration of free LBK leads to a significant prolongation of tail bleeding time, while administration of PNPs/LBK has little effect on the bleeding time. In addition, it is also a good choice to increase the function of responsive drug release on nanoplatelets, such as the use of hydrogen peroxide-responsive platelet membrane-coated nanoparticles for thrombus therapy (Zhao et al., 2021). These studies indicated that the nanoplatelets provide a promising thrombotherapeutic agent, which can effectively target the thrombus site, prolong the internal circulation time, and greatly reduce the hemorrhagic side-effects.

For traditional plasminogen activators, in addition to the hemorrhagic side-effects, there is also the risk of damaging the blood–brain barrier (BBB) and causing neurotoxic effects during ischemic stroke treatment (Su et al., 2008; Niego et al., 2012). In order to obtain the synergistic therapeutic effects provided by thrombolytics and neuroprotectants, a more complex dual-modified nanoplatelet (tP-NP-rtPA/ZL006e) has been developed, which was composed of a neuroprotectant-loaded dextran derivative core and a platelet membrane shell that was conjugated with thrombin-cleavable Tat-peptide-coupled rtPA (Xu et al., 2019). This dual-modified nanoplatelet can be used to sequentially deliver rtPA and the neuroprotectant (ZL006e) in a site-specific manner. After reaching the thrombus site through platelet membrane targeting, the release of rtPA was triggered by the upregulated thrombin, and the Tat peptide exposed *in situ* enhanced the penetration of nanoplatelet across the BBB into the ischemic brain for the site-specific delivery of ZL006e. *In vitro* and *in vivo* evaluation showed that tP-NP-rtPA/ZL006e could significantly improve the anti-ischemic stroke efficacy in rat models, enhance the neuroprotective effect. It would be better if this study could be combined with reducing hemorrhagic function.

In the future, the above-mentioned modification method of nanoplatelets can be used for reference, by pre-processing platelet cells to over-express certain proteins on the platelet membrane, and then transfer the platelet membrane to the surface of polymeric nanoparticle to obtain more functionalized nanosystems.

## Conclusion

With the further study of thrombosis mechanisms and the continuous development of bio-nanotechnology, strategies for thrombus treatment have been constantly improved. Polymeric nanocarrier-based delivery systems have been developed to address a series of challenges that are still encountered in thrombolytic drug therapy, and the application of novel biomimetic cell membrane-modified nanocarriers in thrombolytic therapy has also become the focus of current research.

Many factors should be considered with regard to the design of nanocarrier-based drug delivery systems, such as the collocation and encapsulation between thrombolytic drugs and polymeric nanocarriers, the specific targeting function and responsive release function of the nanosystem, as well as the biocompatibility and biosafety. Utilizing the modifiability of polymer nanoparticles, a variety of targeting molecules (cRGD and heparin, etc.) can be modified simultaneously on polymer nanoparticles, while targeting integrin, P-selectin, and fibrin to improve the targeting accuracy of thrombolytic drugs. Moreover, the drug utilization and therapeutic effect can be improved by various responsive drug release modifications, such as shear-stress response modification on the surface of polymer nanoparticles. In addition, some corresponding imaging diagnosis and other functions can also be considered.

Unlike most tumors, thrombus sites are located in the bloodstream, so the thrombolytic agent can more easily reach the pathological target *via* the circulatory system without crossing layers of barriers. Consequently, it is more necessary for the antithrombotic nanosystems to have stronger targeting and adhesion characteristics, be able to target and bind into the thrombus site in the bloodstream, and achieve deep penetration.

In addition, there are still many areas that can be improved in order to advance nanocarrier-based thrombus treatment technology from the laboratory to clinical practice. Since thrombosis occurs at different locations and at varying development levels among different patients, it is possible to explore the utilization of fluorescent imaging, photoacoustic imaging and other diagnostic methods to determine the thrombosis development stage, and carry out corresponding personalized treatment, realize the integration of diagnosis, and treatment in the same nanosystem. If a platelet membrane-modified nanocarrier system is used, the patient’s own platelets can be used as the membrane source to achieve individualized treatment. Moreover, leveraging the facile design of biomimetic polymer nanosystems to strengthen the synergetic treatment of multiple therapies will help to combine the advantages of various thrombolytic strategies, enhance their active targeting capabilities, and reduce the undesirable side-effects, such as hemorrhagic risk and neurotoxicity of thrombolytic therapy.

In conclusion, although extensive research has been conducted in recent decades, the translation of thrombolytic therapy from the experimental research stage to clinical applications still faces many challenges. With the continuous optimization of nanomaterials and the rapid advancement of nanotechnology, more innovative and efficient biomimetic polymeric nanocarrier-based systems can be anticipated, which will provide versatile platforms and opportunities for significant advances in antithrombotic therapy.

## Author Contributions

QG reviewed the literatures and wrote the manuscript. HD revised and finalized the manuscript. All authors contributed to the article and approved the submitted version.

## Funding

This work was supported by the National Natural Science Foundation of China (No. 21871180), the Tracking Program for Professor of Special Appointment (Eastern Scholar) at Shanghai Institutions of Higher Learning (No. SHDP201802), the Science and Technology Commission of Shanghai Municipality (Nos. 18520710300 and 18JC1413500), and the Open Project of Translational Medicine of SJTU (No. TMSK-2021-108).

## Conflict of Interest

The authors declare that the research was conducted in the absence of any commercial or financial relationships that could be construed as a potential conflict of interest.

## Publisher’s Note

All claims expressed in this article are solely those of the authors and do not necessarily represent those of their affiliated organizations, or those of the publisher, the editors and the reviewers. Any product that may be evaluated in this article, or claim that may be made by its manufacturer, is not guaranteed or endorsed by the publisher.
